# The Influence of Electromagnetic Pollution on Living Organisms: Historical Trends and Forecasting Changes

**DOI:** 10.1155/2015/234098

**Published:** 2015-02-25

**Authors:** Grzegorz Redlarski, Bogdan Lewczuk, Arkadiusz Żak, Andrzej Koncicki, Marek Krawczuk, Janusz Piechocki, Kazimierz Jakubiuk, Piotr Tojza, Jacek Jaworski, Dominik Ambroziak, Łukasz Skarbek, Dawid Gradolewski

**Affiliations:** ^1^Department of Mechatronics and High Voltage Engineering, Gdansk University of Technology, Własna Strzecha Street 18A, 80-233 Gdansk, Poland; ^2^Department of Electrical Engineering, Power Engineering, Electronics, and Control Engineering, University of Warmia and Mazury, Oczapowskiego Street 11, 10-736 Olsztyn, Poland; ^3^Department of Histology and Embryology, Faculty of Veterinary Medicine, University of Warmia and Mazury, Oczapowskiego Street 13, 10-719 Olsztyn, Poland

## Abstract

Current technologies have become a source of omnipresent electromagnetic pollution from generated electromagnetic fields and resulting electromagnetic radiation. In many cases this pollution is much stronger than any natural sources of electromagnetic fields or radiation. The harm caused by this pollution is still open to question since there is no clear and definitive evidence of its negative influence on humans. This is despite the fact that extremely low frequency electromagnetic fields were classified as potentially carcinogenic. For these reasons, in recent decades a significant growth can be observed in scientific research in order to understand the influence of electromagnetic radiation on living organisms. However, for this type of research the appropriate selection of relevant model organisms is of great importance. It should be noted here that the great majority of scientific research papers published in this field concerned various tests performed on mammals, practically neglecting lower organisms. In that context the objective of this paper is to systematise our knowledge in this area, in which the influence of electromagnetic radiation on lower organisms was investigated, including bacteria,* E. coli* and* B. subtilis*, nematode,* Caenorhabditis elegans*, land snail,* Helix pomatia*, common fruit fly,* Drosophila melanogaster*, and clawed frog,* Xenopus laevis*.

## 1. Introduction

Current technologies have become a source of omnipresent electromagnetic pollution from generated electromagnetic fields and resulting electromagnetic radiation. In many cases this pollution is much stronger than any natural sources of electromagnetic fields or radiation. Wireless and radio communication, power transmission, or devices in daily use such as smartphones, tablets, and portable computers every day expose people to electromagnetic pollution. The harm caused by this pollution is still open to question since there is no clear and definitive evidence of its negative influence on human beings. This is despite the fact that extremely low frequency electromagnetic fields were classified as potentially carcinogenic. For these reasons, in recent decades a significant growth can be observed in the scientific research on the influence of electromagnetic fields and/or electromagnetic radiation on living organisms.

Electromagnetic fields and/or electromagnetic radiation, as electromagnetic pollution, affect various elements of the environment. Among the elements of that environment all living organisms should be placed at the first position. Therefore it becomes very important to appropriately determine the nature and related side effects of electromagnetic pollution and its impact on living organisms. Every day living organisms are exposed to different types of electromagnetic pollution. However, all of them can be well characterised by their physical parameters such as type (electric, magnetic, electromagnetic), frequency, and intensity/power. Electronic devices such as smartphones, tablets, microwave ovens, radio, and television sets emit low intensity electromagnetic radiation at frequencies from 300 MHz to 300 GHz that can be associated with microwaves. On the other hand power transmission lines and electric devices are strong sources of electromagnetic fields (primarily electric for power transmission lines, primarily magnetic for transformers, or electromagnetic for antennas) and radiation of much lower frequencies but much higher intensities.

According to the European Commission the sources of nonionizing electromagnetic radiation can be classified as [1]:

- radio frequency fields (RF fields),
- intermediate frequency fields (IF fields),
- extremely low frequency fields (ELF fields),
- static fields.

In order to illustrate quantitatively the authors' considerations presented above, the most typical sources of electromagnetic fields and/or electromagnetic radiation that influence living organisms are listed and described in Table 1.

It should be realised that different types of electromagnetic fields and/or electromagnetic radiation are responsible for different types of phenomena that can be observed as a result of radiation exposure.

For example, high energy microwave radiation at frequencies from 300 MHz to 300 GHz can be carcinogenic and cause thermal effects, increasing the temperature of exposed organisms. On the other hand the same type of microwave radiation at lower frequencies from 100 kHz to 300 MHz has no such effect. It is very important to note that the sources of electromagnetic radiation characterised by field frequencies below 300 GHz can be associated with the nonionizing type of radiation [2].

On the other hand low frequency electromagnetic fields are the source of another type of electromagnetic radiation as in the case of power transmission lines or transformers (by the action of the processes and devices present in the Power System [3]). Such electromagnetic fields that are characterised by field frequencies of 50 Hz or 60 Hz are quasi-stationary and their two field components (electric and magnetic) can be considered as separate [2].

The opinions of researchers about the influence of electromagnetic pollution on living organisms are divided. This is due to the fact that earlier studies very ambiguously indicated either negative or positive, or sometimes neutral, influence of electromagnetic fields and/or electromagnetic radiation. The scale of this problem can be illustrated by the fact that from 1980 to 2002 more than 200 epidemiological studies were published about the effects of electromagnetic fields generated by power transmission lines on human beings. About 60% of them indicated no negative effects of these fields, whereas the remaining 40% reported some smaller or greater negative effects caused [2, 4].

For these reasons in recent decades a significant growth can be observed in the scientific research effort to understand the influence of electromagnetic fields and/or electromagnetic radiation on living organisms. Alarming reports of potentially harmful effects of electromagnetic pollution drew the attention of the World Health Organization (WHO), which in 2007 presented a summary report of an international research program titled Electromagnetic Fields [5]. In that program more than 1,100 various scientific publications and research reports were examined. In the report section dedicated to the effects of low frequency magnetic fields of 50 Hz and 60 Hz it was stated that there are no firm grounds to tighten up the current limits for long-term exposure to these fields; however, caution is advised [5]. In May 2011, in Lyon, France, the International Agency for Research on Cancer (IARC) and WHO qualified the electromagnetic fields of radio frequencies as possibly increasing the risk of developing a malignant brain cancer,* glioma*, which is mainly associated with the use of mobile phones [6].

**Figure 1:**
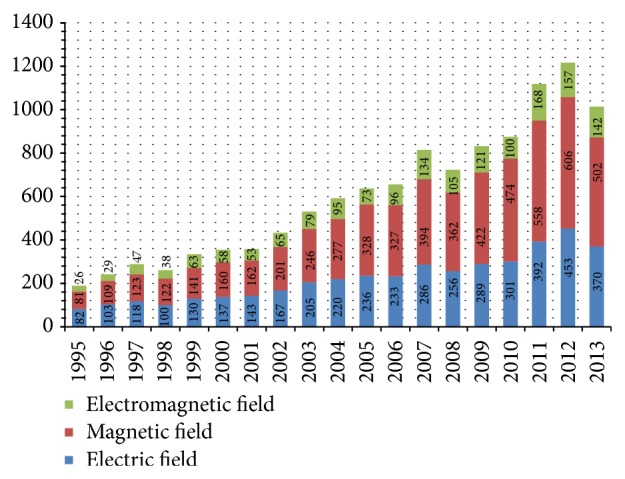
The annual number of research papers published on the influence of electromagnetic fields and/or electromagnetic radiation on living organisms, based on* ScienceDirect* database.

The problems described above still remain unanswered today and result in a great increase of interest in all aspects of electromagnetic pollution and especially its influence on living organisms. This statement can be also backed up by Figure 1, which presents the annual number of research papers published after 1995 and entirely dedicated to this problem, based on the Science Direct research publication database. The following list of keywords was used for the search of related publications: influence, electromagnetic radiation, magnetic field, electric field, and living. The search results include both epidemiological and experimental studies.

## 2. Methods and Materials

In general, research on the influence of electromagnetic fields and/or electromagnetic radiation on living organisms reported in the available literature can be either epidemiological (described in detail in Section 3.1) or experimental (described in detail in Section 3.2).

Epidemiological research concerned the observation of human individuals who had been exposed to increased electromagnetic radiation for longer periods of time, such as railway workers or people living in the neighbourhood of power transmission lines. On the other hand experimental research concerned specific selected model organisms and as such were conducted considerably more often than epidemiological studies. In contrast to the epidemiological research, in the experimental research the appropriate selection of model organisms is always of the greatest importance and must be completed prior to any experimental phase and is also based on the nature of the research and expected results.

In this paper, research results reported in the available literature, focused on specific and current investigations concerning model organisms, are presented and discussed. In this context the paper may be considered as offering certain guidelines for those who want to start research in the area of electromagnetic fields and/or electromagnetic radiation and their influence on living organisms.

**Figure 2:**
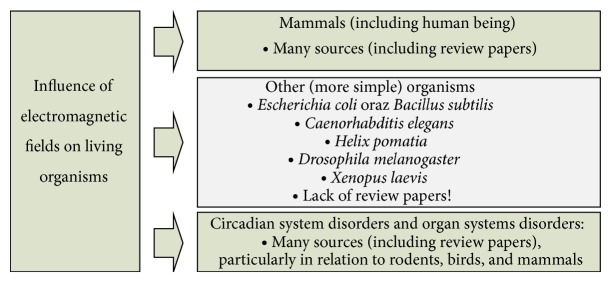
Three main thematic groups distinguished based on the analysis of research papers and reports available in the subject literature.

The review carried out by the authors in this paper was based on important research papers and reports available in* IEEE Xplore Digital Library*,* ScienceDirect*,* PubMed,* and* Google Scholar* databases. Two criteria were used in order to differentiate the results taken into consideration: epidemiological and experimental. At the same time three main thematic groups can be easily distinguished based on the careful analysis of the selected papers and reports; please see Figure 2.

- A group of research papers and reports on the influence of electromagnetic fields on mammals also including human beings: this is also the largest group and it comprises research results of epidemiological and experimental nature as well as review papers. However, review papers are not the subject of the current authors' analysis.
- A group of research papers and reports on the influence of electromagnetic fields on lower organisms such as bacteria, nematodes, molluscs, arthropods, and amphibians: within this group numerous papers and reports are focused on several specific model organisms, which are the species of bacteria* Escherichia coli* and* Bacillus subtilis*, nematode* Caenorhabditis elegans*, land snail* Helix pomatia*, common fruit fly* Drosophila melanogaster,* and clawed frog* Xenopus laevis*.
- The extensive results of research related to the influence of electromagnetic fields on the model organisms just mentioned until today have not been a subject of a unified and thorough review analysis. This is the main objective of the current author's analysis.
- A group of research papers and reports on the influence of electromagnetic fields on rhythm abnormalities and functioning of various systems (mostly immune) in the case of different animal species, especially rodents, birds, or mammals: the effects of electromagnetic radiation on the functioning of the pineal gland were investigated both epidemiologically [7–11] and experimentally [12–35]. Within this group most papers and reports concerned the influence of electromagnetic fields on birds and were carried out on chickens* Gallus gallus *subsp*. domesticus* and Japanese quails* Coturnix coturnix *subsp*. japonica* [34–39]. It should be noted that both the chicken species have practically the same number of genes as humans, which is from 20,000 to 23,000 for the chickens and from 20,000 to 25,000 for humans. Moreover, the firm position of the chickens in scientific research has economic grounds, since the global consumption and production of chicken meat and eggs constantly increase [40]. Also the influence of electromagnetic fields on immune systems of rats and humans was investigated and reported in [37, 41–43].
- As before, these types of problems are not the subject of the current authors' analysis, since it was extensively and systematically analysed by the authors in their other review paper published in 2014 [44].

## 3. Field Influence on Living Organisms

### 3.1. Epidemiological Investigations: Historical Review

Historically, the 19th century was the golden age for electricity and magnetism and the time of their rapid development as scientific branches. What is more, at those times the opinion about a positive influence of electricity and magnetism of the human body, to be found in many medical textbooks, was very common [2]. However, in the 50s and 60s of the 20th century this positive opinion started to gradually evolve and change as a response to new discoveries that followed the development of relevant areas of contemporary science. Many scientists who tried to describe the mechanisms of the phenomena observed were more often inclined to the opinion of a neutral effect of electricity and magnetism on living organisms [2].

One of the first reports of their potentially harmful effects on living organisms was an epidemiological research report published in 1979 by Wertheimer and Leeper [45]. They examined the health of children from Denver (Colorado, USA), who lived in homes exposed to magnetic fields of high intensities. The intensities of the magnetic fields under consideration were estimated based on the total number of power transmission lines nearby the homes exposed and based on the total number of other lines transmitting electrical energy in the investigated area [45]. It was stated by the authors that the children exposed to higher intensity magnetic fields had slightly higher risks of developing leukaemia than unexposed children. The authors also developed in a visual way their own methodology to estimate the exposure level based on the total number of all transmission lines in the area of residence. The published results raised a lot of controversy mainly because of the methodology that was used by the authors, which omitted and neglected many other important effects. Nevertheless, they resulted in an increased interest of scientists in this area of investigation.

It should be also said that later tests confirmed the validity of the charges against the report of Wertheimer and Leeper. The health examination of the children of Rhode Island [46] excluded connections between the influence of magnetic fields and an increased level of morbidity to cancer. However, at the same time different results were obtained and published by researchers from Sweden [47], who found that the incidence of leukaemia may decrease contrary to the incidence of brain tumours, which may increase, in the case of human individuals exposed to higher intensity magnetic fields [47].

The National Institute of Environmental Health Sciences of United States of America [48] proposed a new methodology to be used in this kind of examination. While Wertheimer and Leeper [45] in their report used information from children's death certificates, the new methodology was meant to be based on information from children's health cards. Despite this, negative effects of higher intensity magnetic fields on the children's health could not be confirmed. However, at the same time certain alarming results were published by the same researchers from Sweden [49], who studied all children under the age of 16, who lived within the span of 25 years from 1960 to 1985 no closer than 300 meters from 220 kV or 400 kV power transmission lines [49]. The authors noticed the incidence of diagnosed leukaemia in this group of children which was 2.4 times greater than among their peers. Quite different results were obtained by researchers from Denmark [50] and Finland [51], who could not formulate any similar conclusions under similar test conditions, and who found no direct magnetic field influence on the children's health.

A significant contribution to the clarification of the influence of electromagnetic fields on living organisms, based on the extensive analysis of the existing research results, was made independently by two research groups: Ahlbom et al. [52] from Stockholm and Neutra et al. [53] from Los Angeles. The use of meta-analysis in order to analyse data from previous research papers and reports allowed the two groups to obtain sufficiently large and representative research material. As a result of their independent and parallel research very similar conclusions were obtained, which allowed them to estimate the safe level of the magnetic field intensity as equal to 0.33 A·m^−1^. Ahlbom et al. [52] and Neutra et al. [53] suggested that magnetic fields of higher intensities than the safe level of 0.33 A·m^−1^ raise the risk of developing leukaemia by a factor of two. However, their research results [52, 53] could not confirm any increased risk of developing other cancer diseases including glioblastoma. It should be emphasized that this safe limit is an estimated value and as such has not been confirmed by any rigorous calculations.

Another very thorough and comprehensive analysis of the influence of magnetic fields generated by high-voltage power transmission lines on the risk of developing cancer in children was conducted by Draper et al. [54]. The authors themselves were very surprised by the results obtained, though they were later confirmed by other researchers from Iran [55], Tasmania [53], and Norway [56]. Draper et al. [54] examined children from England and Wales, who lived within 200 m of high-voltage power transmission lines at birth, having therefore higher risks of developing lung cancer. They also took into consideration children who developed leukaemia, who lived within the distance from 200 meters to 600 meters from the field sources, where typical intensities of magnetic fields were lower than 0.08 A·m^−1^ [54]. It should be realised that such intensities of magnetic fields are significantly lower than the field intensities generated by electrical devices in daily use, such as refrigerators, washing machines, radio, and television sets, which questions the main research hypothesis. Similar inconsistencies could be found in the first epidemiological research report by Wertheimer and Leeper [45] as well as in the reports published [54–57]. However, based on the results presented in [52–54] a new safe level of the magnetic field intensity for children was established as equal to 0.15 A·m^−1^. Yet again this value is only an estimated boundary, which is not sufficiently supported by any rigorous calculations.

The main problem that can be formulated in this kind of research is the determination of the direct field source, to which the subjects under investigation are exposed. The authors of the above-mentioned papers and reports silently assumed that the main sources of electromagnetic fields were always high-voltage power transmission lines, while the contribution from low-voltage parts of the electrical system was neglected. The low-voltage contribution may have its sources in buildings themselves or in their neighbourhood, as, for example, they can be generated by working household appliances such as washing machines, refrigerators, radio, or television sets. All these factors make precise and unique determination of any intensity limit for electromagnetic fields practically impossible, despite the fact that such a limit is strongly required in almost all epidemiological literatures. It should be also said that regardless of their certain drawbacks all the papers and reports already mentioned form the basis of a modern view on the influence of electromagnetic fields and/or electromagnetic radiation on living organisms.

A different and very important area of investigation concerned all groups of professional workers exposed to long-term and/or high intensity magnetic fields. However, the results of research obtained in this case also turned out to be inconclusive. Because of that, in Denmark over 2.8 million adult citizens were examined in order to identify the groups exposed to magnetic fields of intensities stronger than 0.24 A·m^−1^ [50]. In total 154,000 people were classified as temporarily exposed to such intensive magnetic fields and 18,000 as constantly exposed [50]. During the examination 39 cases of leukaemia were confirmed, which suggested the risk of circulatory system cancer 1.6 times greater in comparison to 24 cases noted in a control group. However, no cases of malignant tumours were observed [50]. Meanwhile, totally different results were obtained in Norway, where railway workers were examined, who actively worked in the second half of the 20th century. A comparison of health of steam and electric train service workers and operators suggested no negative influence of magnetic fields, while at the same time the number of diagnosed cancer cases turned out to be smaller [2]. On the other hand in the United States a comprehensive examination of 134,000 workers was performed, who were employed in the power generation sector. The results obtained from the examination confirmed 4,833 cases of cancer [58]. van Wijngaarden et al. exanimated a cohort of 138,905 male electric utility workers from five US companies [59]. They found that a mortality rate from cardiovascular diseases and cancer was higher among those workers who worked close to electric fields in comparison to the administration personnel in the same companies [59].

Other examination results reported and published in the literature were carried out among Swiss railway workers and suggested endocrine system disorders in many cases. The side effects were observed after a 5-day exposure to magnetic fields, where the field frequency was 16.7 Hz [7]. A decreased excretion of melatonin related compounds in the urine was also observed in the case of those workers, who were exposed to magnetic fields of frequencies of 60 Hz [8]. These changes were observed after the second day of their working week.

In order to clarify all the above considerations, a synthetic summary is presented in Table 2.

### 3.2. Experimental Investigations

#### 3.2.1. Selection of Model Organisms

It can be noted from the available literature that the investigation on the influence of electromagnetic fields and/or electromagnetic radiation on living organisms requires more thorough research. In this type of research the appropriate selection of relevant model organisms is of very great importance.

A major contribution to the research of the influence of electromagnetic fields on animals was made by Japanese scientists, who conducted a series of tests in four independent research centres in Japan within the span of 6 years from 1993 to 1999 [2]. In the tests animals were firstly exposed to a constant magnetic field of intensity from 400 A·m^−1^ to 4000 A·m^−1^and then examined for cancer incidences. The negative results obtained were later confirmed by scientists from the U. S. and Germany and clearly proved that strong magnetic fields of low frequencies cause no permanent physiological problems as well as no changes to genetic structures [2].

In January 2013 a very extensive report by the Institute of Environmental Sciences and the National Institute for Public Health in the Netherlands was published [36]. The authors of this report made a very thorough review of all published results related to the environmental effects of electromagnetic fields from RF to EMF within the frequency range 10 MHz to 3.6 GHz. In total, 113 original research papers were selected, mostly focused on birds (embryos and eggs), mycelium, and plants. The authors concluded that, in 65% of all cases (50% for animals and 75% for plants), certain influences of the electromagnetic fields were observed in the cases of large and small doses of radiation. The authors [36] divided the selected research papers into sections by making a careful assessment of the contribution of each section, which involved birds, other vertebrates, insects, plants, and other organisms. The latter group included, among others, bacteria* Escherichia coli*, nematode* Caenorhabditis elegans,* and land snail* Helix pomatia*. The authors also emphasized that the electromagnetic radiation had a significant influence on these organisms; however, they concluded that the results obtained cannot be directly transferred and related to human individuals, not only because of a lack of standardisation procedures, but primarily because of the limited number of observation specimens [36]. Moreover, after a thorough analysis of the most recent results available in the literature they underlined the need to extend this kind of investigation to a larger number and a wider range of specimens [36].

Basic information on the model organisms found in the related literature and discussed in the following parts of this paper (bacteria* E. coli* and* B. subtilis*, nematode* Caenorhabditis elegans*, land snail* Helix pomatia*, common fruit fly* Drosophila melanogaster*, and clawed frog* Xenopus laevis*) is collected and presented in Table 3. As can be clearly seen, the information included in the table strongly supports the need for further scientific investigations that aim to determine the influence of electromagnetic fields and/or electromagnetic radiation on the living organisms mentioned above.

#### 3.2.2. Bacteria* E. coli* and* B. subtilis*

The influence of electromagnetic fields and/or electromagnetic radiation on bacteria* Escherichia coli* and* Bacillus subtilis* has been investigated by many researchers for many years. Essential information about both bacteria can be found in [60]. These two types of bacteria are recognized nowadays as gram-negative and gram-positive model organisms mainly due to their well-identified and documented metabolism, structure, and heredity. The growth of* E. coli* and* B. subtilis* is relatively simple and related costs are low, while at the same time cell division stays around tens of minutes and with identification process of resulting mutations being straightforward [61]. Out of the two bacteria the endospores of* B. subtilis* are not only very easily identified but are also substantially more resistant to adverse environmental conditions [62].

Between the years 1944 and 2013 a great number of research papers were published summarizing results of investigations on the influence of electromagnetic fields on* E. coli* and* B. subtilis* in the area of health care, food protection, and husbandry. In one of the first papers Fleming [63] subjected bacteria* E. coli* to electromagnetic radiation of varied frequencies within the range from 11 MHz to 350 MHz. The results obtained indicated a possibility to inactivate bacteria cells by electromagnetic radiation with no local temperature increase. However, later these results could not be reproduced by Brown and Morrison [64]. Furthermore, a local temperature increase due to electromagnetic radiation was reported by Berdnikova et al. [65]. On the other hand no effects of electromagnetic radiation within the frequency range from 10 MHz to 20 MHz on the bacteria vitality were reported in [66] and no cell inactivation could be repeated at the frequency of 60 MHz [67]. In comparison to these efforts a significant success in the inactivation of bacteria* B. subtilis* was achieved in the case of electrostatic fields of intensity of 15 kV·cm^−1^ by Bu et al. [68]. It should be added that the inability to successfully inactivate bacteria* E. coli* by microwaves was recently confirmed by Hamoud-Agha et al. [69].

In 1967 Goldblith and Wang [70] reported that electromagnetic radiation of high frequencies of 2.45 GHz could interact with both types of bacteria. In their opinion the process of bacteria deactivation was feasible and similar to commonly used temperature treatments [70, 71], while in [72] the resemblance between the dynamics of heat and microwave treatments was described in more detail. In 1968 another research paper on the influence of high frequency electromagnetic radiation on the bacteria was published by Webb and Dodds [73], where* E. coli* metabolism was investigated under electromagnetic radiation of frequency of 136 GHz. During their investigation a slowdown in cell division and suspension of some metabolic processes were observed. A year later it was found that the absorption of electromagnetic radiation of particular frequencies by various cells' walls can result in alterations in important metabolic processes [71]. More recent results published in the literature indicate that observable disorders of bacteria* E. coli* growth can result from electromagnetic radiation of frequencies within the range from 70.6 GHz to 73 GHz [74].

An increased secretion of beta-galactosidase as a result of electromagnetic radiation was described in [71, 75], which was associated by the authors with small variations in temperature at the cellular level. Moreover, in [76] the energy of ELF electromagnetic radiation was characterised as a factor intensifying changes in* E. coli* metabolism induced by a temperature increase. Simultaneously, the bacteria were described as being highly resistant to electromagnetic radiation due to the autoregulation mechanisms of numerous biological processes. In [77] these relationships were also proven; however this was in the case of magnetic fields only. Furthermore, the metabolism acceleration of bacteria* E. coli* was observed and described by German researchers in 1995 [78]. They suggest that the observed increase resulted from the application of high intensity electromagnetic fields above 1.6 kV·m^−1^, while at the same time no difference between various field frequencies was noted. In conclusion it was stated that slight temperature changes at the cellular level are responsible for the bacterium metabolism acceleration. Synthetic information from investigation results on bacteria* E. coli* and* B. subtilis* is collected and presented in Table 4.

#### 3.2.3. Nematode* Caenorhabditis elegans*

More than thirty years ago Sydney Brenner precisely characterised the nematode* Caenorhabditis elegans*, which allowed scientists and researchers to use it as a model organism. More information about* Caenorhabditis elegans* can be found in [79].

Since that time more than 7,000 publications and reports have been published covering all possible aspects of its body functioning starting from social behaviour and ending at neurotransmission [80], while in 1998 the entire genome of* Caenorhabditis elegans* was sequenced [81]. It was also found that about 40% of* Caenorhabditis elegans* genes are in common with human genes, as well as many cellular processes [82]. A great scientific effort was made in order to understand regulation, interaction, and expression of the whole set of genes in the genome of* Caenorhabditis elegans* [83]. As a result new genetic and molecular tools became available for investigation of all relevant subjects.

Additionally it should be said that* Caenorhabditis elegans* as a model organism is characterised by a simple, multicellular structure consisting of exactly 959 cells, comprising a variety of tissues such as muscles and nerves. At room temperature the nematode has a relatively short lifespan of about 3.5 days, during which it is able to deposit from 300 to 1000 eggs [80]. A great advantage of* Caenorhabditis elegans* is its sexual dimorphism, which allows for easy observation of both mutations in the processes of self-fertilization as well as hybridization in the case of mating with a male partner [80]. It is worth noting that* Caenorhabditis elegans* is transparent at every stage of its life, which allows for easy investigation and observation of all phenomena taking place in its body such as mitosis and cytokinesis. Furthermore the entire lineage of every cell in* Caenorhabditis elegans* during its embryonic development and postembryo was also described in detail by Sulston [75, 84]. This fact allows the continuation of investigations and observations of the mutated nematode phenotype at the single cell level [80]. Possible cryopreservation and long-term storage of the nematode for further future examination are also a matter of great significance.

All the factors mentioned above resulted in a high scientific interest in nematode* Caenorhabditis elegans* as a subject of research that aimed to explain various biological issues [80] including primarily the influence of electromagnetic fields and/or electromagnetic radiation on living organisms.

The earliest papers published in the literature regarding* Caenorhabditis elegans* reported on the influence of long-term weak electromagnetic radiation at microwave frequencies within the range from 750 MHz to 1 GHz and power of 0.5 W. Their authors claimed that at an ambient temperature of 25°C this kind of electromagnetic radiation can lead to thermal shock effects [85] in transgenic nematodes. Moreover, in order to achieve a similar thermal reaction with no influence of electromagnetic radiation a higher temperature of 28°C was required. This fact let de Pomerai et al. [85] state that the direct result of microwave radiation on nematodes is a thermal shock. This kind of investigation was continued by scientists from Nottingham [86], who studied the effect of microwave radiation of the same parameters on larvae of* Caenorhabditis elegans*. In order to properly describe the observed changes they defined a number of additional factors such as the growth rate (GR), the size of deposited eggs, and maturing proportion (MP) [86]. As a result of radiation they observed 8% to 11% increase in the growth rate (GR) as well as 28% to 40% increase in the maturing proportion (MP). However, they also found 10% decrease in the growth rate (GR) in the case of the reference population that was subjected only to temperature modulations; nematodes were heated up to 28°C, as indicated in [85], while the maturing proportion (MP) was unaffected.

The observed changes indicate that microwave electromagnetic radiation has a direct influence on* Caenorhabditis elegans* as well as an indirect influence resulting from elevated temperature from active microwaves [66]. Similar investigations concerned electromagnetic radiation of lower frequencies within the range from 300 MHz to 750 MHz. It was observed that microwave radiation of such characteristics, particularly of 750 MHz, increases the stress hormone level [87]. Very similar results were obtained by German researchers [88], who observed the same effects as resulting from the exposure to microwave electromagnetic radiation of frequency of 50 MHz [88]. Synthetic information from investigation results on nematode* Caenorhabditis elegans* is collected and presented in Table 5.

#### 3.2.4. Land Snail* Helix pomatia*

The relative simplicity of the investigation of nervous system responses of land snails as well as their low breeding costs [89] results in the fact that land snailsare commonly considered as model organisms. In the past many different snail species were examined in order to determine their resistance to electromagnetic fields and/or electromagnetic radiation [89–93]. In the case of* Helix pomatia*, of particular interest are research results concerned with resting potential of its nerve cells when exposed to magnetic fields and electromagnetic radiation [91]. In both these cases the authors examined the influence of a low-power magnetic field of intensity 98.5 A·m^−1^ as well as low-power electromagnetic fields of intensities within the range of 55.6 mA·m^−1^ to 2.701 A·m^−1^ and low frequencies within the range of 8.3 Hz to 217 Hz. Only those effects that remain unrelated to local changes in temperature were considered. It was found that hyperpolarization of nerve cells results in changes in resting potential; however, this effect was only observed in the case of electromagnetic fields. This fact led to the conclusion that calcium release from the cell cytoplasm was observed only in the presence of the electrical components of electromagnetic fields [91].

In the case of other land snail species, papers by Regoli et al. [92] and Ossenkopp et al. [93] provide much very valuable information. Regoli et al. [92] investigated the influence of electromagnetic fields of frequency of 50 Hz, which can be associated with power transmission lines, as prooxidant in the case of snail* Helix aspersa*. The research programme spanned a two-month field exposure of the snail under investigation and during that time two different values of the field intensity were tested, 0.596 A·m^−1^ and 2.288 A·m^−1^. As a result significant disorders of oxidation at cellular level, lysosomes membrane damage, and loss of DNA integrity were revealed. Ossenkopp et al. [93] investigated snail* Cepaea nemoralis* under different exposure times within the range of 0.5 hrs to 120 hrs and the magnetic field intensity of 79.43 A·m^−1^. A linear increase of snail mortality was found as a function of the exposure time. Additionally, slight differences between daytime and nighttime exposures were noted. Synthetic information from investigation results on land snail* Helix pomatia* is collected and presented in Table 6.

#### 3.2.5. Common Fruit Fly* Drosophila melanogaster*

Common fruit fly* Drosophila melanogaster* has become one of the most widely used model organisms. The reason for its high position among other model organisms comes from certain of its attributes as a species [94]. The essential principles of its inheritance traits are known nowadays thanks to the pioneering research conducted by Thomas Morgan [94], while various characteristics of* Drosophila melanogaster* as a model organism were described in [95–97].

Thanks to* Drosophila melanogaster* attributes, intensive research on the influence of electromagnetic fields and/or electromagnetic radiation on living organisms could be carried out. However, the published results were very often leading to somewhat ambiguous conclusions.

In 1985 the team lead by Hamnerius observed neither effects of high frequency electromagnetic fields on changes in the eye pigmentation nor genetic changes influencing* Drosophila melanogaster* mortality [98]. On the other hand in 1988 Shima and Tomura observed certain gene changes that affected the wing shape [99], while in 1992 the team led by Ho et al. reported that weak static fields influence* Drosophila melanogaster* during embryogenesis causing changes in its circulatory system [100]. In 1995 Koana et al. described the effect of magnetic fields on the growth of the mitotic recombination frequencies [101]. However, research conducted in 1993 by Kikuchi et al. reported no changes resulting from exposure to electromagnetic fields of extremely low frequencies [102], while Nguyen's team in 1995 found no teratological changes in* Drosophila melanogaster* embryonic cells [103]. However, in the same study they reported that exposure of* Drosophila melanogaster* embryos to the same fields results in the abnormal development of the embryos.

In 2002 Mirabolghasemi and Azarnia investigated the influence of the exposure of eggs and subsequent larval stages of* Drosophila melanogaster* to magnetic fields of intensity of 8.738 kA·m^−1^ and frequency of 50 Hz, with exposure times from 2 hrs to 8 hrs, on the physical form of the adult flies [98]. The examination of morphological characteristics of the adults, such as the head or abdomen, allowed the researchers to state that pathological morphology changes concerned only the adult flies exposed to magnetic fields in the larval stage, whereas field exposure in the egg stage led to no pathological changes. The changes concerned size differences of certain body elements, wing deformation, or even their complete underdevelopment. It is worth noting that the observed pathological changes were also present in the case of control groups but at a lower rate. Additionally, it was noted that the number of pathological cases was directly proportional to the exposure time; however, no significant differences were observed in* Drosophila melanogaster* mortality or gender distribution.

In 2001 the Stamenković-Radak's group conducted a similar investigation under static magnetic fields [104]. In their research the second and the sixth generation of* Drosophila melanogaster* were exposed to a static magnetic field of intensity of 27.8 kA·m^−1^. By measuring some morphological parameters of the adult flies the researchers observed that in later generations the wing size varied for both sexes, though no increased rate of wing asymmetry was noted in comparison with reference groups. They also pointed out that the genes responsible for the size of different body parts of* Drosophila melanogaster* or the development of the wings can have possibly different sensitivities to magnetic fields.

In the era of modern technology human beings are constantly exposed to electromagnetic fields and/or electromagnetic radiation, for example, associated with GSM transmission. Therefore it is not surprising that potential threats posed by this type of electromagnetic radiation on living organisms are of very high interest.

In 2003, a group of scientists led by Weisbrot investigated the effects of electromagnetic radiation associated with GSM transmission on* Drosophila melanogaster* at 900 MHz and 1900 MHz mobile phone transmission frequencies [105]. Separate groups of insects were exposed to electromagnetic radiation daily for 2 hrs over a span of 10 days. This included all stages of* Drosophila melanogaster* development from the egg through subsequent larval stages to the adult fly. As a result a significant increase in the levels of hsp70 protein, SRE bindings, and ELK-1 phosphorylation were observed in the case of exposed larvae. An increased number of mature individuals, up to 50%, were observed. The researchers pointed out that the cause of this effect can be found at the chromosome level as the salivary gland chromosomes of* Drosophila melanogaster* indicated an increased transcriptional activity of 73 out of the 200 transcriptionally active regions.

Similar research was carried out by Panagopoulos et al. [106] involving a group of* Drosophila melanogaster* exposed to alternating magnetic fields generated by a GSM mobile phone transmitting at 900 MHz mode. During the experiment the phone was used in standby and active modes (unmodulated exposure) as well as when receiving and sending text messages (modulated exposure). Measured values of the magnetic field intensity were within the range of 7.943 ± 4.766 mA·m^−1^, for the modulated exposure, and 2.383 ± 0.238 mA·m^−1^, for the unmodulated exposure, and both were considered as safe values. As a result a decline by 50% to 60% in reproduction was revealed for the adult flies exposed to the modulated field and 15% to 20% for the adult flies exposed to the unmodulated fields in comparison to a control group. The authors concluded that exposure lowered the rate of cellular processes occurring during the formation and development of gonads. It seems that this is a result of changes in the cell proliferation rate as well as the rate of DNA, RNA, or protein synthesis.

In 2000 the team led by Kohany carried out research on the influence of electromagnetic fields on 10 000* Drosophila melanogaster* larvae and more than 7 000 adult flies [12]. Selected groups were exposed to electromagnetic fields of 5, 7.3, and 9.38 MHz frequencies and power of about 1 *μ*W. The exposure time varied from 4 hrs to the full fly lifespan. In contrast to previously described tests a Faraday cage was used, screening both investigated and control groups from any interfering fields as well as those used in the experiment. As a result, the reduction of the time of the larval stage by 10% compared to the control group was noted. Another observation was an increase of the adenosine-5′-triphosphate (ATP) to adenosine-5′-diphosphate (ADP) ratio. In the case of the control group the ratio of ATP/ADP was 30% lower than in the case of the test group. No morphological lesions or changes in the lifespan of the adult flies were observed.

In 1995 Koana et al. investigated the influence of magnetic fields on DNA [101]. They examined groups of* Drosophila melanogaster* larvae exposed for 24 hours to a static magnetic field of intensity of 476.6 kA·m^−1^. One group genotype was intentionally mutated. It was observed that the number of adult flies with altered genotype was 8% smaller in the exposed group, but the genotype itself remained unchanged. Based on the results obtained they stated that the larvae DNA code was damaged by the field exposure. As a result of the exposure somatic cells were not able to continue cell division lacking normal code corrective mechanisms, which resulted in an increased mortality. The authors suggested, however, that circumstances under which magnetic fields act directly on DNA molecules causing their damage are unlikely due to the amount of energy required for breaking chemical bonds.

Earlier research [107] carried out by Giorgi et al. proved that* Drosophila melanogaster* exposed to static magnetic field intensities 10 to 12 times greater than the intensity of the Earth's field had a noticeable increased size of their body. It was interesting to note that the increased size persisted in subsequent generations even if they were never exposed to any magnetic field influence. It was also found that the increase was due to the quantity of body cells, which allowed the authors to conclude that static magnetic fields affect the genes that are responsible for their proliferation.

Takashima et al. conducted similar research in 2004 [108]. Groups of* Drosophila melanogaster* to be examined were modified by mei-41D5 mutation inhibiting repair and mei-9A mutation improving the recovery process. The authors discovered that exposure to a magnetic field of intensity of 1.986 MA·m^−1^ and 11.12 MA·m^−1^ and 24 hrs exposure time resulted in a statistically significant enhancement in the frequency of somatic recombination within postreplication individuals with the handicapped repair process. Furthermore, within the remaining individuals the frequency has not changed. These findings suggested that exposure to high density static magnetic fields induces somatic recombination in* Drosophila melanogaster* and that this relation is nonlinear.

In 2000 Graham et al. studied the effects of low frequency magnetic fields on* Drosophila melanogaster* [109] focusing primarily on morphological changes. They observed that magnetic fields of frequency of 60 Hz and intensity of 1.191 A·m^−1^ and 63.55 A·m^−1^ caused a significant decrease in the mass of* Drosophila melanogaster*. Additionally, the individuals that were exposed to the field of a higher intensity of 63.55 A·m^−1^ exhibited lower stability than those exposed to 1.191 A·m^−1^ or than those from the control group. It was surprising to note that the individuals exposed to the field of intensity of 1.191 A·m^−1^ exhibit higher stability than the individuals from the control group. This allowed the authors to conclude that magnetic fields do not always have negative influence. Synthetic information from investigation results on common fruit fly* Drosophila melanogaster* is collected and presented in Table 7.

#### 3.2.6. Clawed Frog* Xenopus laevis*

The African cawed frog* Xenopus laevis* has been used by scientists as a model organism for over 50 years [110]. Despite its relatively long lifespan, which may present a difficulty in laboratory examination, the major advantages of* Xenopus laevis* include the following:

- easy to breed in captivity,
- no special requirements for laboratory conditions,
- high congenital resistance to diseases,
- large number of eggs laid,
- large size of oocytes and embryos enabling easy manipulation and testing.

Years of research on* Xenopus laevis* led to numerous discoveries of many interesting phenomena observable at the microbiological and genetic levels. These, in turn, led to the development of new micromanipulation techniques that enabled observation of microbiological changes. Exact fate mapping, hormonal regulation, study of genetic mechanisms, and accurate identification of transgenesis mechanisms allowed for precise manipulation at the microbiological level leading not only to faster but also to more accurate interpretation of observation results as well as their analysis.

*Xenopus laevis* helped in numerous attempts to investigate the influence of electromagnetic fields and/or electromagnetic radiation of living organisms. In 2010 Severini et al. conducted research on the influence of weak electromagnetic fields on* Xenopus laevis* development capacity [111]. In the experiment tadpoles were exposed to electromagnetic field of frequency of 50 Hz and the intensity within the range from 50.76 A·m^−1^ to 60.69 A·m^−1^ for 60 days. As a result it was observed that the average growth rate of exposed individuals decreased in comparison with a control group. Additionally, it was noted that exposure to the electromagnetic field also accelerated the average time of metamorphosis of tadpoles by 2.4 days.

In 2005, Mietchen et al. [112] examined the influence of strong static magnetic fields on* Xenopus laevis* cerebral cortex. They noted that some of the changes observed could be related to an egg's shell removing procedure, which is performed prior to various subsequent scientific activities. In order to verify if the removal of the egg shell has an important influence they conducted their own experiments examining both processed eggs and unprocessed eggs under static magnetic field of intensity of 7.467 MA·m^−1^. The results of the test revealed changes in pigmentation of the cerebral cortex only in the case of the individuals hatched from the processed eggs, while the pigmentation itself appeared to be dependent on the exposure time. They concluded that pathological changes in pigmentation result from the removal of the egg's shell, the shell seeming to support the formation of the cytoskeletal system as its original purpose.

The concept that electromagnetic fields have no significant effects on the development of* Xenopus laevis* was also confirmed by research carried out in 1995 by a team led by Ueno et al. [113]. During their research they exposed eggs to static magnetic field of intensity of 5.036 MA·m^−1^ for different time intervals. The results of their experiments showed that eggs under continuous 6-hour exposure indicated no significant pathological changes in cell division after transition to tadpoles. Moreover, the same result was observed for an increased 18 hour exposure time but at a reduced intensity level of 3.574 MA·m^−1^. Similar conclusions were reached by Kay et al. [114], who examined the influence of electromagnetic radiation accompanying magnetic resonance imaging (MRI) procedures. Their results proved the absence of morphological and functional changes. Synthetic information from investigation results on clawed frog* Xenopus laevis* is collected and presented in Table 8.

## 4. Conclusions

Based on the review of research results published in the available literature and related to the influence of electromagnetic fields and/or electromagnetic radiation on living organisms the following critical conclusions can be formulated.

1. The available literature provides scattered and ambiguous information about the safety of electromagnetic fields and/or electromagnetic radiation.
2. The influence of electromagnetic pollution on living organisms remains undefined.
3. There are substantial gaps in the present knowledge about the influence of electromagnetic pollution, especially in the case of experimental investigations conducted on animals.

As a consequence of the above-mentioned facts the area of scientific research related to the influence of electromagnetic pollution on living organisms is very popular among scientists all around the world.

One of the key problems in this kind of research is the elimination of secondary radiation sources, which proves to be a difficult task; therefore all epidemiological investigations should be followed by experimental ones. More importantly, despite the extensiveness of the research results available, no clear answers have been given yet to the question of whether electromagnetic pollution has a bad influence on living organisms. Also the answer to the opposite question whether electromagnetic fields and/or electromagnetic radiation can be beneficial to living organisms in certain cases has not been answered so far.

It should be noted that all the relevant model organisms possess certain features useful in order to conduct biological research. These include resemblance to other living organisms, which allows model organisms to be considered substitutes for other organisms, including human beings.

In this paper special attention has been paid to model organisms different from mammals, including bacteria* E. coli* and* B. subtilis*, nematode* Caenorhabditis elegans*, land snail* Helix pomatia*, common fruit fly* Drosophila melanogaster*, and clawed frog* Xenopus laevis*. However, it has been found by the authors that due to restricted frequency spectra investigated, as well as the intensities of electromagnetic field sources, and due to the nature of observed phenomena, the results reviewed by the authors cannot be considered as complete and cannot be extrapolated onto human beings.

So far, research conducted in the area of the influence of electromagnetic fields and/or electromagnetic radiation on living organisms has no comprehensive character, so it is not possible to formulate any relationships between the electromagnetic field characteristics and the field influence. Despite the lack of sufficient empirical data resources, several interesting hypotheses were proposed in the literature, according to which electromagnetic fields affect the pineal gland and its hormone melatonin, interfering with its physiological mechanisms leading to sleep disorders, lower mood, reduced concentration, depression, and the development of certain cancers [115–119]. These hypotheses, unsupported by sufficient scientific evidence reflect explicitly the importance of the pineal gland in investigations of related mechanisms of the harmful effects of electromagnetic fields and/or electromagnetic radiation published in journals, textbooks, and even on the Internet. However, based on current knowledge these claims remain unjustified and require systematic scientific verification.

## Figures and Tables

**Table 1:** Sources of electromagnetic fields and radiation influencing living organisms [1].

Type	Frequency	Source
Static^*^	—	Natural, video screens, magnetic resonance imaging, and other diagnostic/scientific equipment, electrolysis, and welding devices

ELF	Below 300 Hz	Power transmission lines, home wiring, car electric engines, electric trains and trams, and welding devices

IM	300 Hz ÷ 100 kHz	Video screens, antitheft devices used in cars, homes, and shops, card readers, metal detectors, magnetic resonance imaging, and welding devices

RF	100 kHz ÷ 300 GHz	Radio, television, smartphones, tablets, microwave ovens, radar and radio transmitters, and magnetic resonance imaging

^*^Static electromagnetic fields do not exist and should be understood as either static electric or magnetic fields.

**Table 2:** A synthetic summary of a historical review of epidemiological investigations.

	Field source	Examined group	Location, years	Observation and result	Literature
1	MF from power transmission lines	Children	Denver, USA, 1979	Increased risks of leukaemia, objection of lacking precision	[45]
2	MF	Children	Rhode Island	No incidents of cancer	[46]
3	MF	Various	Sweden	Decreased incidents of leukaemia Increased incidents of brain tumour	[47]
4	MF from 220 kV and 400 kV power transmission lines distant <300 m	Children < 16	Sweden, 1960–1985	Incidents of leukaemia increased 2.4 times	[49]
5	MF	Children	Denmark	No incidents of malignant tumour Incidents of leukaemia increased 1.6 times	[50]
6	MF	Adults	Finland	No incident, no bad influence	[51]
7	MF	Children	Stockholm, Los Anglels	Field intensities below 0.33 A·m^−1^ reduce risks of leukaemia 2 times	[52, 53]
8	MF from HV power transmission lines distant >200 m and 200 m ÷ 600 m	Children	England, Wales, Iran, Norway, Tasmania	Safe filed intensity for children equal to 0.15 A·m^−1^ – objection of Lacking precision	[52–57]
9	MF from power transmission lines	Railway workers	Norway	Decreased incidents of cancer	[2]
10	MF from power transmission lines and devices	Power sector workers	USA	Morbidity of cancer increased 1.2 times 3.6% confirmed cancer incidents for field intensities exceeding 3.44 A·m^−1^	[58, 59]
11	MF of 16.7 Hz and 60 Hz	Railway workers	Swiss, USA	Blood generation system disorders	[7, 8]

**Table 3:** Basic information on selected model organisms.

Organism	Description	Literature
*Bacillus subtilis *	(i) Gram-positive bacterium, cell division every 10 minutes (ii) Rod-shaped, around 0.8 *µ*m in diameter and 3 *µ*m in length (iii) Endospores resistant to high temperature (10% alive after 1-hour bath is boiling hot water, 1% after 2-hour bath), high pressure (2 GPa for 45 minutes), space vacuum (for 24 hours) (iv) Responsible for bread ropiness (v) Produces peptide antibiotics (*polymyxin*, *subtilisin*, etc.) and enzymes (*amylase*, *protease*, etc.) (vi) Few literature sources documenting the influence of EMF	[120]

*Escherichia coli *	(i) Gram-negative bacterium, cell division every 20 minutes (ii) Rod-shaped, around 0.8 *µ*m in diameter and 3 *µ*m in length (iii) Element of human and warm-blooded animals colon gut bacterial flora, where it participates in digestive processes and production of B and K group vitamins (iv) Pathogenic leading to human diseases of the digestive or urogenital systems (v) Dies out in temperature of 60°C after 20 minutes, in propitious conditions (for example in faeces) can survive for 1 year (vi) Used for production of a human hormone, insulin (vii) Few literature sources documenting the influence of EMF	[120, 121]

*Caenorhabditis elegans *	(i) Transparent body hermaphroditic nematode (male individuals represent around 0.2% of the entire population), around 1 mm in length (ii) Lifespan of 2 or 3 weeks at room temperature, lifecycle around 56 hours (iii) Body consisting of 959 somatic cells including 302 neurons, internal organs consisting of a constant number of cells (iv) No negative effects of cryopreservation (v) Sole organism of a fully mapped connectome and genome (in 1998) (vi) Numerous body processes similar to human, shares 40% of genes with humans (vii) Few literature sources documenting the influence of EMF	[121, 122]

*Helixpomatia *	(i) Common species of a land pulmonary snail (ii) Lives in the Southeast and Central Europe (iii) Shell 5 cm in diameter (iv) Body cells contain 54 chromosomes (v) Hatching of young snails after 3 or 5 weeks (vi) Considered as a model organism due to simplicity of its nervous system response investigations (vii) Few literature sources documenting the influence of EMF	[89, 123]

*Drosophila melanogaster *	(i) Standard model organism, considered as the essential one (ii) Requires no special laboratory conditions (iii) High fecundity: females laying up to 100 eggs per day and around 2000 in their lifespan (iv) Short lifespan around 10 days at room temperature (v) Body cells contain 4 chromosome pairs (vi) Exhibits sexual dimorphism: females about 2.5 mm, males about 2 mm (vii) About 75% of known genes of human diseases can be matched with the genome of fruit flies (viii) About 50% of protein sequences have mammalian homologs (ix) Moderate number of literature sources documenting the influence of EMF	[121, 124]

*Xenopus laevis *	(i) Model organism for more than 50 years (ii) Easy to breed in captivity, requires no special laboratory conditions (iii) Invasive species capable of surviving droughts hidden in sludge at the bottoms of water reservoirs (iv) Males around 8 cm in size, females around 13 cm in size (v) Lifespan ranges from 5 to 15 years (vi) Females sensitive to human chorionic gonadotropin and in the past used as a natural pregnancy test (vii) Females lay large eggs with large embryos that are easy to manipulate and test (viii) Transparent tadpoles allow observation of subsequent stages of their development (ix) Moderate number of literature sources documenting the influence of EMF	[110]

**Table 4:** Information from investigation results on bacteria *E. coli* and *B. subtilis*.

Type	Parameters	Results	Literature
EMF	11 MHz ÷ 350 MHz	(i) Possibility of inactivation (ii) No local increase in temperature (unconfirmed)	[63, 64]

EMF	10 MHz ÷ 20 MHz, 60 MHz	(i) Local increase in temperature (ii) No effects on the vitality (iii) No inactivation	[64–67]

EF	15 kV·cm^−1^	(i) Significant inactivation in the case of *Bacillus subtilis *	[68]

RF	Microwaves of various frequencies	(i) Possibility of inactivation (ii) Resemblance between the dynamics of heat and microwave treatments (iii) Absorption of the radiation of particular frequencies may affect metabolic processes (iv) Disorders of *E. coli* growth resulted from EMF of 70.6 GHz and 73 GHz (v) Increased secretion of beta-galactosidase (vi) Highly resistant to EMF & MF due to autoregulation of numerous biological processes (vii) No difference between various field frequencies (probably thanks to slight temperature changes at the cellular level)	[69–71, 73–78]

**Table 5:** Information from investigation results on nematode *Caenorhabditis elegans*.

Type	Parameters	Results	Literature
RF	750 MHz ÷ 1 GHz 0.5 W 25°C Long-term exposure	(i) Thermal shock (ii) Increase in the growth rate between 8% and 11% (iii) Increase in maturing proportion between 28% and 40%	[66, 85, 86]

RF	50 MHz, 300 MHz, 750 MHz Long-term exposure	(i) Increase of the stress hormone level	[87, 88]

**Table 6:** Information from investigation results on land snail *Helixpomatia*.

Type	Parameters	Results	Literature
MF, ELM	98.5 A·m^−1^ 55.6 mA·m^−1^ ÷ 2.701 A·m^−1^ 8.3 Hz ÷ 217 Hz	Nerve cells hyperpolarization under electromagnetic fields	[91, 92]

ELM	50 Hz, 0.596 A·m^−1^ and 2.288 A·m^−1^ 2-month exposure	Significant disorders of oxidation at cellular level Lysosomes membranes damage DNA integrity loss	[93]

EF, ELM	79.43 A·m^−1^ 0.5 hrs ÷ 120 hrs exposure	Linear increase in the mortality Slight differences between daytime and nighttime exposures	[93]

**Table 7:** Information on investigation results on common fruit fly *Drosophila melanogaster*.

Type	Parameters	Results	Literature
MF	27.8 kA·m^−1^	(i) Variation in the wing size in later generations (ii) Unaffected wing asymmetry (iii) Varying sensitivity of the genes responsible for the size of different body parts (development of wings)	[104]

MF	476.6 kA·m^−1^ 24 hrs exposure	(i) Reduced by 8% number of mature individuals with altered genotype group (ii) Increased mortality of larvae probably due to MF influencing their DNA code	[101]

MF	397.2 A·m^−1^ ÷ 476.6 A·m^−1^	(i) Noticeable increase of the body size (persisted in later generations under no field influence) (ii) Permanent exposure affects the genes responsible for proliferation	[107]

MF	1.986 MA·m^−1^ ÷ 11.12 MA·m^−1^ 24 hrs exposure	(i) Statistically significant enhancement in frequency of somatic recombination within the postreplication individuals with the handicapped repair process (ii) Nonlinear relation between somatic recombination and field exposure	[108]

EMF	3–30 Hz	(i) No changes in embryonic cells (ii) No teratological changes (iii) Abnormal development of embryos	[98]

EMF	60 Hz 1.191 A·m^−1^ and 63.55 A·m^−1^	(i) Significant decrease in mass (ii) Lower stability than control group as well as group exposed to 1.191 A·m^−1^ (iii) Possible positive field influence	[109]

EMF	8.738 kA·m^−1^ 50 Hz 2 hrs ÷ 8 hrs exposure	(i) Pathological changes in larvae stage exposure (differences in body elements size, wing deformation, complete underdevelopment) (ii) Pathological changes also in control groups but at lower rate (iii) Number of pathological case directly proportional to exposure time	[98]

EMF	900 MHz ÷ 1900 MHz 2 hrs daily for 10 days	(i) Significant increase in the level of *hsp70* protein, SRE bindings, and ELK-1 phosphorylation of larvae exposed (ii) Increased by 50% number of mature individuals (iii) Field exposure may affect chromosomes as the salivary gland, an increased transcriptional activity of 73 of the 200 transcriptionally active regions	[105]

EMF	7.943 ± 4.766 mA·m^−1^ (modulated exposure) 2.383 ± 0.238 mA·m^−1^ (unmodulated exposure) 900 MHz	(i) Decline in reproductive performance by 50% to 60% for individuals exposed to modulated fields (ii) Decline in reproductive performance by 15% to 20% for individuals exposed to the unmodulated fields (iii) Field exposure affects females more than males (iv) Lowered rate of cellular processes occurring during formation and development of gonads (v) Changes in cell proliferation rate, rate of DNA, RNA, or proteins synthesis	[106]

**Table 8:** Information on investigation results on clawed frog *Xenopus laevis*.

Type	Parameters	Results	Literature
EMF	50.76 A·m^−1^ ÷ 60.69 A·m^−1^ 50 Hz, days of exposure	(i) Decreased averaged growth rate in comparison to control group, decrease from 0.48 step/day to 0.43 step/day (ii) Accelerated average time of metamorphosis of tadpoles by 2.4 days	[111]

MF	7.467 MA·m^−1^	(i) Changes in pigmentation of the cerebral cortex in the case of removed egg shells (ii) Pigmentation of a function of the exposure time (iii) Pathological changes in pigmentation	[112]

MF	5.036 MA·m^−1^ various exposure periods	(i) No pathological changes after continuous 6 hrs exposure time of eggs in cell division after transitioning to tadpoles (ii) Similar observations for 18 hrs exposure time to less intensive filed of 3.574 MA·m^−1^ (iii) Absence of morphological, functional changes, and the timing of development abnormalities in tested individuals	[113, 114]
